# Postdoctoral employment and future non-academic career prospects

**DOI:** 10.1371/journal.pone.0278091

**Published:** 2022-12-01

**Authors:** Johannes König

**Affiliations:** Institute of Economics and INCHER, University of Kassel, Kassel, Hessian, Germany; Lund University: Lunds Universitet, SWEDEN

## Abstract

Most recipients of doctorates leave universities some years after graduation, while little is known about their future non-academic career prospects. I report results from a novel microlevel database that links information about doctoral dissertations completed in Germany with doctorate recipients’ social security records. The results, based on graduates’ individual careers for five broader subject groups, indicate that postdoctoral periods do not result in a wage premium when changing to the non-academic sector.

## Introduction

A higher level of education is often associated with higher individual income. This might be one reason why an increasing number of individuals go to university [1] after leaving school. Currently, one in two secondary school graduates starts a bachelor’s program [2], and compared to the 1990s in many developed countries, such as the USA [3], Germany [4], and Sweden [5], an increasing number of students began a doctorate after graduation.

The link between educational times in school and bachelor’s and master’s degrees on later income is well documented [2, 6–10]. In contrast, information on further qualification phases after the doctorate is scarce. Current research often solely focuses on a few years after graduation or STEM subjects [11, 12].

The meaning of monetary returns for retention in academia after completing the doctorate is crucial. Phases after the doctorate, such as remaining at the university as a postdoc, are seen as qualification phases for individual careers. This argument is often used to justify the comparatively insecure working conditions for doctoral researchers and postdocs at universities [13, 14]. However, higher education systems in many developed countries train and employ far more doctoral graduates and postdocs in the short term than can be employed at universities and non-university research institutes in the long term [15]. The personnel structure at universities and public laboratories resembles a pyramid with an often almost inexhaustible supply of motivated students, doctoral students and postdocs, on the one hand, and only a few permanent positions for senior researchers at the top, on the other hand [16]. Temporary employment contracts are intended (e.g., in Germany) to ensure that universities continue to have enough vacancies for the future qualification phase [13], as well as produce graduates who are also available to the economy as a well-trained workforce. Therefore, it is not surprising that many university graduates (including those with doctorates [17, 18]) leave the university sometime after graduation. In the case of the USA, the National Science Board shows that only a minority of the 41% of all PhDs in science and engineering work in the academic sector. Many doctorate recipients work in the private sector or for the government [19]. In Germany, only 14% of all doctorate recipients are employed at university, while the majority find employment in the private sector [20]. Descriptive analysis indicates that a year after completing the doctorate, 46% of doctoral graduates with previous employment at a university or non-university research institute have already left academia. This proportion rises to 66% 5 years after graduation.

At the individual level, there are many reasons why recipients of doctorates change sectors. Not all of them leave due to a lack of prospects for permanent university positions. Even if there is often a clear preference for an academic career, as the Nature 2017 PhD survey reveals [21], interest in non-academic careers often increases for doctoral students the more time they stay in the academic system [22], and a majority of postdocs change career goals during their postdoctoral experience [23]. Outside the university system, recipients of doctorates are sought-after workers because of their abstract problem-solving ability [24], their transferrable skills [25] and their contribution to (industrial) innovations [26, 27], while high levels of education in a region are shown to have a positive impact on regional productivity [28].

This paper analyses, based on social security records of doctorate recipients from graduation years 1994 to 2009, how staying at a university or non-university research institute after completing a doctorate affects doctorate recipients’ income 5 years after graduation when leaving the academic sector. This is a relevant research question, as a large number of recipients of doctorates stay for at least some years after receiving their doctorate in the university system. Only a minority succeed in obtaining permanent positions in academia [20, 29, 30], and the majority leave the academic sector in the short and medium term. I use Germany as an empirical example. Germany has one of the highest numbers of trained doctoral graduates in the world [2] and a long tradition of employing scientists outside of the classical academic sector [31, 32]. Research is performed on the 5 largest subject fields, namely, humanities and arts, social sciences, science and mathematics, medicine, and engineering. The results indicate that the link between education time and income established by Mincer [6] does not apply to the most highly qualified. For none of the subject groups considered is a significant positive link found between postdoctoral periods and income. In the aggregate, the results suggest that doctoral graduates who remain at the university earn significantly less income in the medium term than those who leave the university directly after graduation. A matching approach is used to reduce selection bias towards retention in a university career.

The paper is structured as follows. First, a brief overview of the employment situation of doctoral graduates outside academia is given, with a particular focus on Germany. This section is followed by a discussion about the impact of education on earnings, taking into account the selection problem regarding different educational paths. Then the data and the identification strategy is presented, as well as the results of the empirical estimation. Finally, the results are discussed, considering their importance for higher education policy.

## Qualification periods and labour market outcomes

### The employment of doctorate recipients outside of the academic sector and the institutional setting in Germany

By educating undergraduate and postgraduate students, universities not only train their own future employees but also a well-trained workforce for the economy. Many developed countries have a long tradition of employing researchers in the private sector. In Germany, this tradition goes back to the 19th-century dye industry. Companies in the chemical industry, such as BASF, Bayer and Höchst, recognised early the potential of graduates and scientists as highly qualified personnel for their industrial research [31]. This development in Germany served as a blueprint for many other countries, such as the USA, where doctoral graduates also significantly contributed to industrial research at the end of the 19th century [32].

In the mid-20th century, the need for a highly educated workforce for the economy increased. Higher education, once available only to an elite circle, now became accessible to broad portions of society [1]. Similar to many other European countries, numerous new universities were founded in Germany in the 1960s to 1980s [33] to meet the demand of the economy for highly qualified workers [34, 35].

Currently, among all OECD countries, approximately 50% of individuals under the age of 25 are enrolled in higher education. A total of 1.4% of the population under 30 starts a doctorate, with Germany’s share of 2.8% being approximately twice the OECD average and roughly equal to that of the United Kingdom [2].

Not surprisingly, many doctoral graduates in developed countries such as the USA [19] or Germany [20] find employment in the private sector after graduation. The majority of doctorate recipients in Germany leave the university within the first few years after graduation, with only a fraction of all doctoral graduates remaining at universities in the long term [36].

A special feature of German universities in this respect is their flexibility in awarding fixed-term employment contracts. Special legal regulations on fixed-term employment for researchers at universities were introduced in a specific law in 1985 (HRG §57a-57 f). This allowed for the temporary employment of academic staff for various reasons, such as professional training and third-party funding. In the 2000s, these statutory regulations on fixed-term employment in science were changed, and a maximum period of 12 years was introduced by *Wissenschaftszeitvertragsgesetz* (up to 6 years before the doctorate and 6 years after a completed doctorate), whereby there are numerous special regulations, e.g., for parenthood and in the field of medicine [13]. Temporary employment in the public sector serves to increase staff turnover and permits responding flexibly to changing conditions [37]. At universities, this fluctuation in personnel is intended to ensure that a correspondingly large number of vacancies also exist for the next generation of qualified researchers [38], whereby the legal regulation is at the same time intended to give "junior" researchers a sufficiently long time for their qualification work before and after the doctorate [39].

### Qualification periods and income

Overall, a positive influence of an individual’s qualifications on later work success, especially on income, has been found in numerous studies. A focus of past research was on the influence of the number of school years and bachelor’s or master’s degrees on later income, but interest in research on postdocs is growing [11, 12, 40, 41].

Mincer [6] showed that a substantial proportion of the differences in individuals’ incomes can be explained by differences in time in education across individuals. A recent comparison of the incomes of workers with different levels of education across OECD countries also shows that the incomes of workers with tertiary education exceed those of workers who have attained upper secondary education [2].

One challenge in determining the influence of different qualification levels on later career success (especially for higher education) is the endogeneity of these decisions. In the case of (self-) selection into different career paths, the causal effect of education level on labour market success can no longer be determined.

Several identification procedures are proposed in the literature to deal with the endogeneity problem [42]. Instrumental variable approaches have often been used, where researchers use an observable covariate that affects the level of education attained but is not correlated, e.g., with different abilities. For example, Angrist and Krueger [43] and Staiger and Stock [8] use the date of birth as an instrument for years of schooling, where individuals born earlier in the year are older at entry and, thus, have less schooling than students born later in the year.

Another branch of research is devoted to siblings and twins [7, 9, 10]. Here, it is assumed that some of the unobservable heterogeneity that distorts the analysis of the impact of education on later earnings is reduced by looking at individuals with the same family background.

In addition, there are various matching approaches [44, 45], which try to reproduce the conditions of an experiment ex post. A control group is sought for the treatment group with certain educational attainment/years of education that are as similar as possible to the treatment group in terms of observable variables. Here, evidence, especially for higher education, is mixed. Rzepka [46], for example, uses propensity score matching to compare the career outcomes of those who enrol in higher education compared to those who pursue careers based on vocational training. Despite considerable uncertainty, individuals with a university degree achieve a higher cumulative income than those with only vocational training. Lutschek and Zwick [47] match individuals with tertiary vocational education (*Meister* or *Techniker*) and academic education on the basis of individual characteristics to make these groups comparable in their decision for higher education and their earnings potential. They show that those who have completed an academic education as a supplement to their apprenticeship earn significantly less than those who have completed a supplementary tertiary vocational program such as *Meister* or *Techniker*. Regarding research on postdoctoral education, evidence for selected subjects shows that when controlling for selection bias for STEM [40], science and engineering [11] and biomedicine graduates [12] postdoctoral programs did not increase earnings.

## Empirical approach

### Database on doctorate recipients

This paper uses data from the IAB-INCHER project of earned doctorates [IIPED] to investigate the link between further employment at universities and non-university research institutes on later labour market outcomes for 5 broader fields of study. This new microlevel dataset combines data on published doctoral theses from the catalogue of the German National Library (DNB) with the Integrated Employment Biographies (IEB). It allows a longitudinal view of the career paths of doctoral graduates across different graduation years, the time dedicated to their careers and sectors. The use of social security data is legally regulated in Germany by § 75 SGB X. The author received permission for this project by the German Federal Ministry of Labour and Social Affairs.

Due to the publication requirement for dissertations, the catalogue of the German National Library contains a copy of almost all dissertations published at German universities for the period under observation. The Integrated Employment Biographies (IEB) contain detailed information on the employment histories of all employees who are subject to social security contributions and/or have marginal income, social security recipients, jobseekers, unemployed persons and participants in measures. The IEBs are based on process data from the Federal Employment Agency. In total, approximately 80% of the workforce in Germany is covered, whereby self-employed persons and civil servants are not included. Both datasets were linked using machine learning methods [48].

The research paper analyses how staying at a university or non-university research institute after a doctorate affects doctorate recipients’ income when leaving the academic sector. Doctorate recipients’ income is analysed at the end of December, 5 years after graduation. The analysis considers only those doctoral graduates who were employed at a university in the year of graduation and up to 5 years after graduation but who are no longer employed at a university or non-university research institute at the end of the 5-year period after graduation. Due to this selection criterion, doctoral graduates will be excluded from the analysis if there is more than one year between the end of a doctorate recipients’ employment contract at a university or non-university research institution and the submission of the dissertation. This excludes industrial doctorates (where it is uncertain whether they were ever truly interested in an academic career), doctoral students whose funding expired before completion of the doctorate and who did not find an extension contract as well as doctoral candidates seeking employment outside academia at an early stage. This has the advantage that a relatively homogeneous group of doctorate recipients is considered who either leave the university in the year of completing their doctorate or up to 5 years after graduation. This reduces the risk that distorting selection effects might have on the analysis. There are various reasons why employment contracts of doctorate recipients at universities or non-university research institutes end before graduation. However, some of these plausible reasons speak against including doctoral graduates who left the university well before receiving their doctorate as a reference group for the analysis of postdoc periods on income. This sample selection also means that the results presented cannot be regarded as representative of all doctoral graduates who choose a position outside academia later. However, the presented empirical findings can be considered representative for doctorate recipients who were employed as postdocs at universities for up to 5 years after graduation and later changed the employment sector.

The sample is limited to doctoral graduates who were older than 20 years and younger than 45 years at the time of graduation and to doctoral graduates who graduated between 1994 and 2009. This ensures that the careers of doctoral graduates can be analysed for 16 graduation years and that the analysis is not solely driven by macroeconomic shocks such as the global economic crisis. In addition, the analysis is limited to doctoral graduates in full-time employment due to a lack of information on working hours for part-time employment.

### Variables

The main variable of interest is the logarithmised daily income received in the non-academic sector 5 years after completing the doctorate. This period was deliberately chosen to minimise the influence of the statutory regulation of *Wissenschaftszeitvertragsgesetz*, which allows universities to temporarily employ doctorate recipients in most fields of study up to 6 years after completion of the doctorate.

Since data are collected for administrative purposes, income is right-censored in the IEB. Income is only reported up to the income threshold of the social security statistics. This value is adjusted annually and amounted to €72,600 for West Germany in 2015. The imputation procedure of Gartner [49] was used to impute the wages. The salary was adjusted for inflation and is reported in 2015 euros.

The main variable of interest is the last year in which a doctorate recipient was employed at a university or non-university research institution for more than 50 days before changing sectors (Nbr_postdoc_years). Because the data included several contracts that did not end in December but continued for a few days in January and ended thereafter, the 50-day cut-off was used to smoothen the data. This cut-off ensures that a doctorate recipient who has a contract at a university that runs only a few days into the year after graduation is not assigned to the next year. A move back to a university or non-university research institution is very rare [36]. For example, of those doctorate recipients who worked in the private sector 4 years after graduation, only approximately 1% were (re)employed at a university or non-university research institution in year 5 after graduation.

The statistical classification of economic activities in the European Community was used to identify universities and non-university research institutes. For non-university research institutes, I use WZ 2008 code 72.11.0, 72.19.0 and 72.20.0. A record linkage completed this information where additional information on non-university research institutions was taken from the *Bundesbericht Forschung und Innovation* (BuFI). In this regularly recurring report, the Federal Ministry of Education and Research (BMBF) has been reporting on the state of the research and innovation system in Germany since the 1960s. It contains information about non-university research institutions and their locations in the respective reporting years. For universities, I use WZ 2008 code 85.42.1 85.42.2 85.42.3. This information was completed by a record linkage based on a complete list of universities in Germany taken from the key index for student and examination statistics from *Destatis*. As a result, the classification used here contains a nearly complete list of all universities and non-university research institutions in Germany. This includes both research institutes that are part of a university as well as independent non-university research institutes. For more details see Koenig [17]. It should be noted that this assignment is based on the employer and therefore cannot reflect the perceived research proximity. For example, it cannot be ruled out that some doctoral graduates continue to take on research-related tasks in another sector or have the "feeling" of working as a scientist or scholar even after leaving the university or non-university research institution. In addition, there may be a few doctoral graduates who remain at the university after receiving their doctorate, but who do not work on a research-related task or do not have the "feeling" of working as a scientist or scholar.

For personal characteristics, I control for gender, German nationality, and age. A dummy is also used to indicate whether the graduate had an apprenticeship before graduation.

There are 5 different dummies used for aggregated subject groups. These are humanities and arts, social sciences, science and mathematics, medicine, and engineering. The classification is based on the classification used by the German Federal Statistical Office (Destatis) [50] and includes all major subject categories. Aggregated subject categories of agricultural sciences and sports are excluded due to low case numbers.

As regional control variables, 3 different dummies are used for different region types (*BBSR Siedlungsstrukturelle Regionsgrundtypen*), as well as a dummy for employment in western Germany. In addition, 10 dummies regarding *Klassifikationen der Berufe 2010 [51]* are used for different occupational fields in which doctoral graduates work.

Different types of work experience may have different effects on subsequent salaries 5 years after graduation. First, I control for the number of years a doctorate recipient has worked before the year of graduation. Second, a variable counts the number of years a doctorate recipient has worked within and after the year of graduation. These two variables together control for the general work experience that doctorate recipients have gained by the end of the observation period. In the Mincer equation, tenure (work experience with an employer) may be similarly important as the general work experience that individuals have accumulated. Recipients of doctorates who left academia directly after graduation may have a longer tenure within their later employers than those who changed professions after some postdoctoral years. I control tenure as the number of years a person has worked at the place of employment at the end of the observation period (5 years after graduation).

In addition, future non-academic career prospects for doctorate recipients can differ depending on their academic employer. Germany has a large number of non-university research institutions with various disciplinary focuses and links to basic as well as applied research. Employment relationships at non-university research institutions such as the Max Planck Institutes or Fraunhofer Institutes are also subject to the statutory regulation of *Wissenschaftszeitvertragsgesetz*. The hiring requirements, duration and profile of qualification positions at non-university research institutions after the doctorate are comparable to those positions of *Nachwuchsgruppenleiter* and junior and tenure-track professorships at universities (see BuWiN 2021 for an overview [20]). At the same time, however, a slightly increased number of permanent employment contracts can be observed at non-university research institutions compared to universities, which may indicate a higher chance of a permanent position at these institutes and a higher adverse selection of researchers who leave the institute within the first years after their doctorate. To control for the influence of the academic employer, a dummy variable was created that takes the value 1 if the last employment in academia was at a non-university research institution.

One reason for staying in research after completing the doctorate may be the lack of employment opportunities outside academia and the ease of obtaining a postdoc position. Dummy variables for the year of graduation are used to control for time-specific effects. Dummy variables for the university granting the doctoral degree are used to control for university-specific effects. These variables also capture university- and time-specific push and pull factors for an academic career. I further use the number of professors and the inflation-adjusted amount of third-party funding per professor at the degree-granting university in the graduation year as indicators of the ease of obtaining a research position. Regional unemployment in the university region in the year of the doctorate serves as an indicator of the lack of opportunities to obtain employment outside the university, while high levels of third-party funding might be correlated with good employment possibilities within the graduation university. These variables are not available for all graduation years under consideration and are, therefore, used in a subsample analysis as a robustness check.

A description of the variables and the summary statistics can be found in S1 and S2 Tables.

### Identification strategy

This paper uses 1-to-1 nearest neighbour matching to reduce observable differences between doctoral graduates who leave the university immediately in the graduation year and those who remain employed at the university as postdocs.

The Stata command psmatch2 [52] was used to identify the doctorate recipients’ nearest neighbour. To determine the impact of postdoctoral retention on salary, individuals who left university in one specific postgraduate year were matched without replacement with one individual who left university in the graduation year.

Exact matching was carried out for the variables subject, gender and year of graduation. The exact matching based on graduation year and subject is intended to ensure that both the group of doctoral graduates who left university directly after graduation and the group of those who left university up to 5 years after graduation are influenced by the same macroeconomic factors, such as business cycle or industry-specific shocks. These shocks and business cycles might influence the opportunities to find an adequate position outside academia and the ease of getting a postdoc position. Within these strata, propensity score matching was performed for the variables age, German citizenship, apprenticeship and previous work experience. In addition to personal variables, this also takes into account that previous work experience and vocational training may influence the choice to remain in academia.

The matching was carried out separately for each treatment level (*Nbr_postdoc_years*). For better comparability, mean, standard deviation, t-tests, and standardised difference in % for the different treatment groups (1 year to 5 postdoc years) are considered aggregated.

Table 1 shows the differences in the variables used for matching. t-tests and standardised difference in % are reported to assess quality [44]. The standardised difference for all matching variables is less than 5%, a level at which the matching is often considered successful in the literature [53].

**Table 1:** Descriptive statistics of matching criteria.

	Nbr_postdoc_years = 0		Nbr_postdoc_years>0		t test	Standardised
VARIABLES	Mean	(SD)	Mean	(SD)		difference in %
Humanities/Arts	0.0156	0.1238	0.0156	0.1238	0.0000	0.000
Social Sciences	0.1086	0.3111	0.1086	0.3111	0.0000	0.000
Natural Sciences/Math	0.5190	0.4997	0.5190	0.4997	0.0000	0.000
Medicine	0.1798	0.3841	0.1798	0.3841	0.0000	0.000
Engineering	0.1770	0.3817	0.1770	0.3817	0.0000	0.000
Graduation year	2000.5860	0.0427	2000.5860	0.0427	0.0000	0.000
Female	0.1974	0.3980	0.1974	0.3980	0.0000	0.000
Age	36.1697	2.6098	36.3044	2.8911	-3.7603	-4.892
German	0.9668	0.1791	0.9664	0.1802	0.1810	0.236
Apprenticeship	0.1914	0.3934	0.1945	0.3958	-0.6099	-0.794
Years worked before graduation	3.8708	2.0517	3.8215	2.2973	1.7376	2.261
Observations	11,816		11,816			

**Fig 1:**
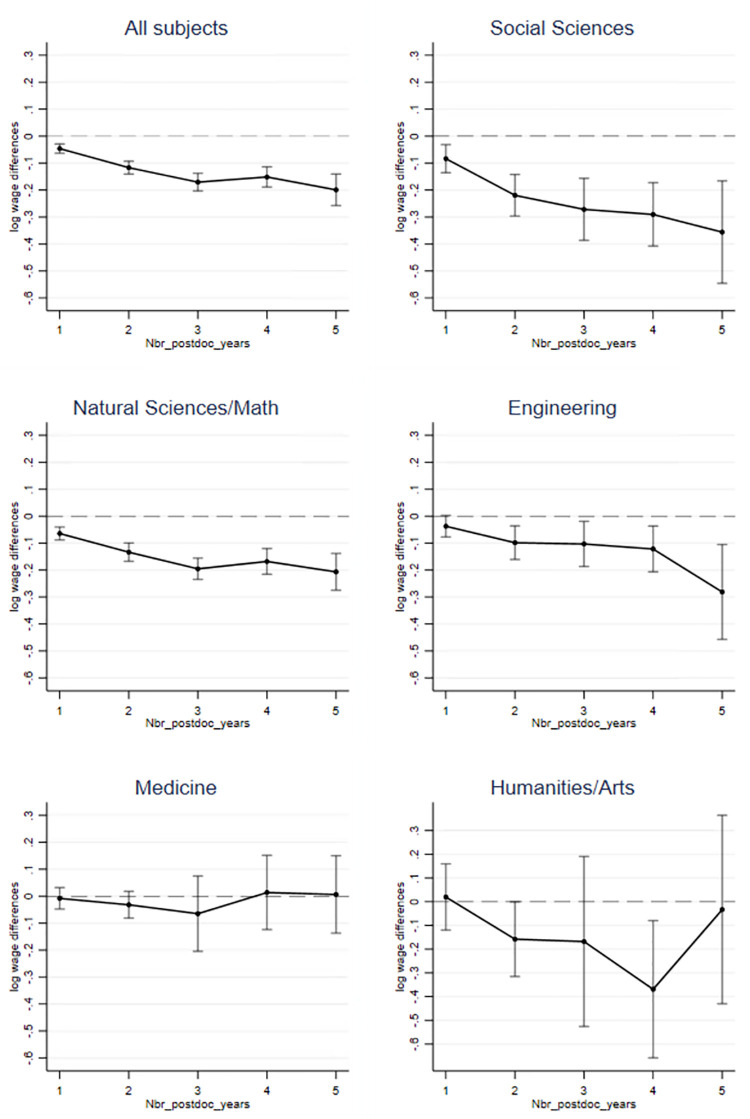
Earnings differences in relation to retention in science.

Linear regressions were then applied to the matched sample to trace the link between postgraduate time in the university system and doctorate recipients’ medium-term career outcomes (Model 1). To investigate subject-specific differences, the models are estimated separately for different broad fields of study (Model 2 to Model 6). To facilitate interpretation, the estimated marginal effects for Model 1 to Model 6 in Table 2 with their 95% confidence interval of the variable *Nbr_postdoc_years* are plotted in Fig 1.

**Table 2:** OLS regression of doctorate recipients’ daily log imputed income 5 years after graduation.

	(1)	(2)	(3)	(4)	(5)	(6)
	ALL	Humanities/Arts	Social Sciences	Natural Sciences/Math	Medicine	Engineering
VARIABLES	OLS	OLS	OLS	OLS	OLS	OLS
Ref. Nbr_postdoc_years = 0	-	-	-	-	-	-
Nbr_postdoc_years = 1	-0.0461***	0.0198	-0.0835***	-0.0641***	-0.0080	-0.0372*
	(0.0087)	(0.0710)	(0.0266)	(0.0123)	(0.0202)	(0.0204)
Nbr_postdoc_years = 2	-0.1168***	-0.1578**	-0.2193***	-0.1334***	-0.0318	-0.0983***
	(0.0122)	(0.0801)	(0.0393)	(0.0173)	(0.0253)	(0.0318)
Nbr_postdoc_years = 3	-0.1704***	-0.1678	-0.2714***	-0.1951***	-0.0648	-0.1030**
	(0.0167)	(0.1821)	(0.0588)	(0.0202)	(0.0712)	(0.0427)
Nbr_postdoc_years = 4	-0.1514***	-0.3691**	-0.2903***	-0.1677***	0.0136	-0.1215***
	(0.0191)	(0.1471)	(0.0600)	(0.0245)	(0.0702)	(0.0432)
Nbr_postdoc_years = 5	-0.1991***	-0.0331	-0.3559***	-0.2065***	0.0064	-0.2811***
	(0.0299)	(0.2018)	(0.0970)	(0.0349)	(0.0733)	(0.0898)
Female	-0.2565***	-0.2039***	-0.2463***	-0.2378***	-0.2596***	-0.3100***
	(0.0098)	(0.0603)	(0.0276)	(0.0139)	(0.0192)	(0.0366)
Ref. Male	-	-	-	-	-	-
Age	-0.0078***	0.0046	-0.0113**	-0.0163***	0.0147***	-0.0045
	(0.0017)	(0.0114)	(0.0049)	(0.0026)	(0.0039)	(0.0041)
German	0.0458**	0.1459	-0.0370	0.0318	0.0567	0.0847**
	(0.0193)	(0.1633)	(0.0915)	(0.0256)	(0.0477)	(0.0417)
Ref. Foreign	-	-	-	-	-	-
Apprenticeship	0.0393***	0.0544	0.0192	0.0172	0.0725***	0.0119
	(0.0099)	(0.0933)	(0.0272)	(0.0169)	(0.0199)	(0.0203)
Ref. No Apprenticeship	-	-	-	-	-	-
Years worked before graduation	0.0116***	0.0277*	0.0172**	0.0129***	-0.0106**	0.0208***
	(0.0025)	(0.0148)	(0.0068)	(0.0039)	(0.0053)	(0.0057)
Years worked after graduation	0.1028***	0.1314***	0.1092***	0.1070***	0.0950***	0.0784***
	(0.0042)	(0.0302)	(0.0138)	(0.0057)	(0.0091)	(0.0127)
Years worked same operation	0.0047*	-0.0125	-0.0102	0.0003	0.0275***	0.0039
	(0.0024)	(0.0193)	(0.0071)	(0.0035)	(0.0059)	(0.0055)
Last employment in non-university research institute	0.0444***	0.1589**	0.0486	0.0613***	-0.0168	0.0644***
	(0.0089)	(0.0787)	(0.0310)	(0.0120)	(0.0212)	(0.0235)
Ref. Last employment at university	-	-	-	-	-	-
Humanities/Arts	-0.2374***					
	(0.0281)					
Social Sciences	0.0601***					
	(0.0174)					
Natural Sciences/Math	-0.0504***					
	(0.0130)					
Engineering	0.0144					
	(0.0163)					
Ref. Medicine	-					
Constant	4.9677***	4.3503***	4.9415***	5.1676***	4.5273***	5.0341***
	(0.0938)	(0.4998)	(0.2870)	(0.1587)	(0.2031)	(0.2514)
Graduation year dummies	YES	YES	YES	YES	YES	YES
Degree-granting university dummies	YES	YES	YES	YES	YES	YES
Regional controls	YES	YES	YES	YES	YES	YES
Dummies occupational field	YES	YES	YES	YES	YES	YES
						
Observations	23,632	368	2,566	12,264	4,250	4,184
R-squared	0.155	0.382	0.219	0.143	0.156	0.163

Robust standard errors in parentheses

*** p<0.01

** p<0.05

* p<0.1

A series of robustness analyses were performed. These aim to determine the stability of the estimates to uncertainties in the dependent variable (inflation-adjusted imputed logarithmised daily income) and sample composition (Model 7 to Model 16). It is important to mention that, overall, doctorate recipients earn a comparably high income 5 years after graduation. At the end of year 5 after graduation, 57% of all doctorate recipients earn an income above the contribution limit to the social security system, which makes them among the top earners in Germany. The censoring of the wage variable for those graduates involves the risk of potential measurement errors due to wage imputation. I use different alternatives of the income variable to investigate how sensitive the results are to the imputation measure used.

First, a dummy variable is used in place of imputed salary in a logit regression indicating whether doctorate recipients have earnings above the contribution limit to social security. The contribution limit is adjusted annually, depending on the development of wages in Germany. While the deflated salary serves as an indicator of the spending capacity of the doctorate recipients, this dummy variable serves as an indicator of where an individual is located in the wage distribution relative to all employees. This dummy variable for high income does not underlie the risks of potential measurement errors of the imputation methods.

In addition, instead of regressions with imputed earnings, a censored-normal regression is estimated for the deflated censored wage. This regression accounts for the fact that some observations are censored as well as uncensored and that the threshold varies over time and region. All regressions are also estimated separately with non-logarithmised income variables (except for the regression with the dummy for high income).

To control for potential biases of sample composition, regressions are not only estimated on data based on 1-to-1 matching but are also presented for the full dataset without matching.

In addition, the future career prospects of doctorate recipients who have been working at universities and non-university research institutions may differ (see above), and this effect might not fully be captured by a dummy variable indicating the respective last employer type. As a further robustness analysis, the full dataset is therefore analysed separately for those whose last postdoctoral academic employment was at a university and those whose last employment was at a non-university research institution (Model 17 to Model 18, S3 Table).

Further, for the subsample of doctorate recipients who graduated after 1998, I control for additional push and pull factors regarding a job inside academia by the regional unemployment rate, third-party funding by professors, and the number of professors in the year of graduation by degree-granting university. The subsample analysis is necessary since data are not available for all years under consideration (Models 19 to 28, S4 Table).

## Postdoctoral employment and later earnings

An OLS regression is used to estimate the daily income of doctoral graduates 5 years after graduation (see Table 2). The main variable of interest here is the last year in which a doctorate recipient was employed at a university or non-university research institution. The estimation is based on the sample in which each doctoral student who left the university in one specific year after completing his or her doctorate was matched to the nearest neighbour based on observables who left the university directly in the year of graduation. Model 1 presents the estimates for all doctoral graduates, and Models 2 to 6 present the estimates for individual subject groups.

On average, women with doctorates earn a significantly lower daily wage than their male counterparts. It is noteworthy that a lower salary for women is also present in the subsample estimations for different subject groups (Models 2 to 6). Interestingly, the wage gap can not only be explained by the different subject choices by women compared to male doctoral graduates.

Work experience before and after the dissertation has a positive effect on medium-term earnings for almost all subject groups. A wage penalty for older students can be found in almost all subject groups (except humanities and arts and engineering), while foreign graduates earn significantly less than native doctorate recipients in the field of engineering.

Further doctorate recipients whose last employment was at a non-university research institute have, on average, a significantly higher income 5 years after graduation compared to doctorate recipients whose last employment before changing employment sector was at a university.

A comparison between the individual subject categories (Model 1) also shows significant differences in income between the different subject groups. The field of medicine serves as the reference group here. Doctorate recipients in the humanities and arts earn the lowest daily salary 5 years after graduation. Doctoral graduates in the social sciences earn the highest daily salary, while the coefficient for engineering is insignificant. Predictive margins indicate that these figures correspond to a daily salary of €237 5 years after graduation for those who left university directly after graduation. There are substantial differences for graduates from humanities and arts (168€), social sciences (275€), natural sciences and mathematics (246€), medicine (217€), and engineering (286€).

The “relatively” low salary in the field of medicine can be attributed, among other things, to their training as specialists. Physicians in Germany earn their doctorates at a relatively young age, and this specialisation within the medical profession usually lasts five to six years. The high income of doctoral graduates in the social sciences can be explained by the large number of graduates who work in management positions after receiving their doctorate.

Fig 1 suggests that there is no wage premium for postdoctoral periods. For example, doctorate recipients (Model 1) earn, on average, 5% less when they leave their academic employer one year after graduation compared to those who leave immediately after graduation. For doctorate recipients who leave academia 5 years after graduation, the wage loss is 18%. The salary losses of doctorate recipients who left academia in the fifth year after receiving their doctorate range from 19% in natural sciences and mathematics, 25% in engineering, and 30% in the social sciences. These numbers should be interpreted with caution, however, as doctorate recipients who leave academia in year 5 after graduation have only spent a short time with their new employers, and the income may be reduced by agreements during the probationary period. It is even more astonishing, however, that significant differences are already evident for most subjects who left academia 1 to 2 years following graduation. Doctorate recipients in the field of medicine are the only subject group for which no significant effect can be found for short- and medium-term stays in academia on later income. It is also striking that none of the models shows a significant positive influence of short- to medium-term stays at a university after graduation on salary 5 years after graduation.

It should be noted that the lack of a significant effect of most years of retention for doctorates in the humanities and arts should be interpreted with caution. Doctorate recipients in humanities and arts earn by far the lowest salaries of all fields 5 years after graduation. Even for those doctorates from the humanities and arts who leave the academic sector relatively late, their salaries are substantially lower than those of all other subject categories.

Table 3 aims to determine the robustness of the results with respect to the matching approach and uncertainties in the imputed logarithmised daily wage variable. The explanatory variables equal them in Model 1 in Table 2. Here, logistic regressions, OLS regressions and censored linear regressions are estimated. Income is in logarithmic and non-logarithmic form, and estimates are shown based on the matched dataset and the full dataset. Robustness analyses regarding the uncertainty of the income variable support the previous results. All models indicate that staying in the university in the short and medium term is associated with wage losses when switching sectors. In this case, wage losses are substantially higher for graduates who left the university relatively late after finishing the doctorate.

**Table 3:** Robustness analysis for regression of doctorate recipients’ income 5 years after graduation.

	(7)	(8)	(9)	(10)	(11)	(12)	(13)	(14)	(15)	(16)
	High Wage (dummy)	Log daily wage (imputed)	Daily wage (imputed)	Daily wage (censored)	Log daily wage (censored)	High Wage (dummy)	Log daily wage (imputed)	Daily wage (imputed)	Daily wage (censored)	Log daily wage (censored)
	Logit	OLS	OLS	Cnreg	Cnreg	Logit	OLS	OLS	Cnreg	Cnreg
VARIABLES	Matched Sample	Matched Sample	Matched Sample	Matched Sample	Matched Sample	Full Sample	Full Sample	Full Sample	Full Sample	Full Sample
Ref. Nbr_postdoc_years = 0	-	-	-			-	-	-		
Nbr_postdoc_years = 1	-0.3043***	-0.0461***	-14.1689***	-6.8091***	-0.0561***	-0.3233***	-0.0465***	-13.2556***	-7.6805***	-0.0631***
	(0.0356)	(0.0087)	(3.0197)	(0.8889)	(0.0083)	(0.0331)	(0.0081)	(2.8064)	(0.8258)	(0.0077)
Nbr_postdoc_years = 2	-0.6125***	-0.1168***	-34.7285***	-14.8291***	-0.1234***	-0.5840***	-0.1095***	-31.5424***	-14.3932***	-0.1191***
	(0.0497)	(0.0122)	(3.9061)	(1.1922)	(0.0112)	(0.0442)	(0.0109)	(3.4636)	(1.0590)	(0.0098)
Nbr_postdoc_years = 3	-0.8526***	-0.1704***	-44.2115***	-20.7249***	-0.1749***	-0.6677***	-0.1243***	-32.5649***	-16.2344***	-0.1336***
	(0.0661)	(0.0167)	(5.4529)	(1.5346)	(0.0149)	(0.0493)	(0.0123)	(3.9487)	(1.1489)	(0.0109)
Nbr_postdoc_years = 4	-0.8216***	-0.1514***	-38.4655***	-20.5726***	-0.1663***	-0.7464***	-0.1269***	-33.8247***	-17.0077***	-0.1361***
	(0.0784)	(0.0191)	(6.2678)	(1.7896)	(0.0165)	(0.0545)	(0.0130)	(4.1948)	(1.2301)	(0.0113)
Nbr_postdoc_years = 5	-1.1090***	-0.1991***	-50.3240***	-24.5145***	-0.2191***	-1.0282***	-0.1608***	-38.1251***	-21.6382***	-0.1855***
	(0.1160)	(0.0299)	(8.6804)	(2.6054)	(0.0267)	(0.0736)	(0.0180)	(5.7237)	(1.5967)	(0.0155)
Constant	0.3838	4.9677***	75.9821**	167.8156***	5.1217***	0.0655	4.8600***	56.6615**	166.7752***	5.1135***
	(0.4287)	(0.0938)	(30.8671)	(6.9780)	(0.0661)	(0.3500)	(0.0769)	(24.2349)	(5.9334)	(0.0562)
Individual controls	YES	YES	YES	YES	YES	YES	YES	YES	YES	YES
Work experiences controls	YES	YES	YES	YES	YES	YES	YES	YES	YES	YES
Graduation year dummies	YES	YES	YES	YES	YES	YES	YES	YES	YES	YES
Degree-granting university dummies	YES	YES	YES	YES	YES	YES	YES	YES	YES	YES
Regional controls	YES	YES	YES	YES	YES	YES	YES	YES	YES	YES
Dummies occupational field	YES	YES	YES	YES	YES	YES	YES	YES	YES	YES
Observations	23,632	23,632	23,632	23,632	23,632	33,396	33,396	33,396	33,396	33,396
(Pseudo) R-squared	0.151	0.155	0.098	0.045	0.160	0.155	0.154	0.097	0.047	0.165

Robust standard errors in parentheses

*** p<0.01

** p<0.05

* p<0.1

As an additional robustness check, the dataset was split into graduates who changed the sectors of employment from a university and those who changed sectors of employment from a non-university research institution (S3 Table). These subsample analyses provide additional information on the extent to which the career prospects of postdocs might differ between those who changed sectors from different academic employers. The results are qualitatively comparable to previous findings. For botch, doctoral graduates who switch from an employment at a non-university research institution and for those who switch from a university employment after postdoctoral periods, wage losses can be observed in comparison to those who leave the university directly in the year of completing their doctorate. It is interesting to note that these wage reductions for those who switch employment sectors from a non-university research institution are comparatively low.

S4 Table (Models 19 to 28) additionally controls for further push and pull factors into an initial academic career. The structure of the table is similar to that of Table 3. In addition to Table 3, S4 Table further controls for the regional unemployment rate in the year of graduation in the university region as well as the amount of third-party funding per professor and the number of professors. Overall, the results support previous analysis, that postdoctoral periods are not associated with higher level of income when changing employment sector.

## Discussion and conclusion

When discussing education and qualifications, people normally consider the number of years in school, their studies with degrees such as bachelor’s or master’s, or even their doctorate. In most of the cases, the qualification phase that follows these academic experiences, such as a postdoc, is not considered. However, one important question is how these advanced qualification phases are linked to individual careers.

The research in this paper is based on 5 broader subject groups and 16 graduation years. Empirical findings suggest that there is no positive link between retention at a university or non-university research institution after receiving a doctorate and later earnings when switching sectors. Postdoctoral periods are associated with individual wage losses that are statistically and economically significant for most subjects.

In addition to the relevance of these results for doctorate recipients’ individual career decisions, these results may also have relevance for higher education policy. Insecure working conditions in academia are often explained by higher education policy that requires employees to meet qualification work goals. This is one reason why they are accepted by many academics. Individuals often hope for better employment opportunities in academia but also in other sectors with a higher education. This research, however, raises the question of whether this correlation in terms of income and higher education also exists for this highly qualified group. In a country such as Germany, where additional periods spent in academia after receiving the doctorate officially count as qualification periods and where a well-established transfer of academics to industry exists, postdoctoral periods do not seem to pay off from a financial point of view. There is also growing evidence from other countries that staying in academia and postdoctoral periods for STEM graduates are not associated with higher incomes. Research has shown that this is the case for French [11] and American postdocs [12, 40, 41]. At the same time, the probability of obtaining a permanent position at a university is very low. In Germany, 92% of full-time academic and artistic staff at universities (under 45 years of age, excluding professors) are employed on fixed-term contracts [20] and only approximately 3% of all doctoral graduates receive a professorship in the long run [20]. In the USA, many postdocs wait long periods for a permanent position, with only a small proportion receiving a tenure-track position [29, 30].

In terms of higher education policy, this research raises the question whether the current employment situation of doctorate recipients in academia can truly be justified by additional individual qualification goals (and the associated positive career prospects) or whether it must be ensured that, in the end, scientists do not pay to be scientists [54].

One limitation of this research is that the utilized matching approach reduces, but may not eliminate the selection bias towards an academic career. This can be particularly problematic when doctorate recipients, who leave the university at various times after graduation, differ in their skills and abilities, which would also reflect differences in later income. Data used for this empirical analysis are based on detailed labour market information. It is reasonable to expect that some of the used matching and control variables like work experience, vocational training, tenure, and age, also control in part for heterogeneity in skills and abilities relevant for doctorate recipients later non-academic occupation. At the same time, however, it must be borne in mind that these variables are only imperfect indicators. This raises the question of who, in the presence of selection bias, could leave the university directly after graduation and earn a substantial higher income in the medium term. One explanation would be that at least some of the ablest doctorate recipients recognise attractive alternatives outside academia early on. A further academic career is only one option for those with a doctorate. Universities compete for capable minds with employers from other sectors. Accepting an attractive job offer from industry directly after the doctorate could be a tempting alternative to years in temporary positions as a postdoc at a university.

In this case, it would be important to find good career prospects for the most capable academics at an early stage to avoid a possible brain drain from science to other sectors. However, further research is needed to fully understand the relationship between further qualification phases after the doctorate and long-term career outcome. Here, especially survey data can be relevant to learn more about the individual motivation of researchers to leave academia at different times in their careers.

## Supporting information

S1 Table Description of variables. (DOCX)

S2 Table Summary statistic. (DOCX)

S3 Table Subsample analysis for doctorate recipients working at university or non-university research institute. (DOCX)

S4 Table Robustness analysis for additional push and pull factors for staying in academia after graduation. (DOCX)
