# Supplementary material for: Postdoctoral employment and future non-academic career prospects
Source: PLoS One. 2022 Dec 1;17(12):e0278091. doi: 10.1371/journal.pone.0278091 (PMC9714870; doi:10.1371/journal.pone.0278091)
Supplement: S1 Table — (DOCX) [file pone.0278091.s001.docx]

Table S 1 Description of variables

|  |  |
| --- | --- |
| VARIABLES | Definition |
| Daily wage (imputed) | Imputed daily wage deflated in 2015 Euro (dependent variable). |
| Log daily wage (imputed) | Logarithmised imputed daily wage deflated in 2015 Euro (dependent variable). |
| Daily wage (censored) | Censored daily wage deflated in 2015 Euro (dependent variable). |
| Log daily wage (censored) | Logarithmised censored daily wage deflated in 2015 Euro (dependent variable). |
| High Wage (dummy) | Dummy, doctorate recipient has a wage above the contribution limit for social security. Regarding to rounding errors the contribution limit is deducted by 2 Euro. |
| Nbr_postdoc_years | Dummy for the last year a doctorate recipient (Nbr_postdoc_years = 0) or postdoc (Nbr_postdoc_years>0) was employed at a university or non-university research institute for more than 50 days. |
| Female | Dummy, doctorate recipient is female. |
| Age | Continuous, age of doctorate recipient at 31 December 5 years after graduation. |
| German | Dummy, doctorate recipient has German nationality. |
| Apprenticeship | Dummy, doctorate recipient did an apprenticeship before graduation. |
| Years worked before graduation | Continuous, years worked before the year of graduation. |
| Years worked after graduation | Continuous, years worked within and after year of graduation. |
| Years worked same operation | Continuous, years doctorate recipient worked in the same operation he was working 5 years after graduation. |
| Humanities/Arts | Dummy, doctorate recipient graduated in humanities/arts. |
| Social Sciences | Dummy, doctorate recipient graduated in social sciences. |
| Natural Sciences/Math | Dummy, doctorate recipient graduated in natural sciences or mathematics. |
| Medicine | Dummy, doctorate recipient graduated in medicine. |
| Engineering | Dummy, doctorate recipient graduated in engineering. |
| Last employment in non-university research institute | Dummy, last academic employer before changing the employment sector was a non-university research institute. |
| Third party funding by prof (defl.) | Continuous, amount of third-party funding (in 1000 €) divided by number of professors deflated in 2015 Euro at degree-granting university in year of graduation. |
| Nbr. professors degree granting university | Continuous, number of professors at degree-granting university in year of graduation. |
| Unemployment rate in university region | Continuous, unemployment rate in university region in year of graduation. |
| Individual controls | Controls for female, age, German and apprenticeship as well as subject groups Humanities/Arts, Social Science, Natural Science/Math, Medicine, and Engineering |
| Work experiences controls | Controls for years worked before graduation, years worked after graduation, years worked same operation, and last employment in non-university research institute |
| Graduation year dummies | Dummies for different years of graduation. |
| Degree-granting university dummies | Dummies, for university awarding doctorate recipients’ degree. Small universities were attributed to category “other”. |
| Region controls | Dummies for three regional types of work region: Agglomerations, urbanised regions and rural regions. German classification of settlement structures (*Siedlungsstrukturelle Regionstypen*) was used. Dummy for employment in western Germany. |
| Dummies occupational field | Dummies regarding to *Klassifikation der Berufe 2010* for 10 different occupational fields in which doctoral graduates work. |
