# Supplementary material for: Postdoctoral employment and future non-academic career prospects
Source: PLoS One. 2022 Dec 1;17(12):e0278091. doi: 10.1371/journal.pone.0278091 (PMC9714870; doi:10.1371/journal.pone.0278091)
Supplement: S2 Table — (DOCX) [file pone.0278091.s002.docx]

Table S 2 Summary Statistic

|  | (1) | (2) | (3) | (4) | (5) |
| --- | --- | --- | --- | --- | --- |
| VARIABLES | N | mean | sd | min | max |
| Log daily wage (imputed) | 23,632 | 5.470 | 0.600 | 2.308 | 7.588 |
| Daily wage (imputed) | 23,632 | 285.1 | 198.7 | 10.05 | 1,975 |
| High Wage (dummy) | 23,632 | 0.573 | 0.495 | 0 | 1 |
| Daily wage (censored) | 23,632 | 170.3 | 30.52 | 13.22 | 200.8 |
| Log daily wage (censored) | 23,632 | 5.110 | 0.279 | 2.581 | 5.302 |
| Nbr_postdoc_years = 0 | 23,632 | 0.500 | 0.500 | 0 | 1 |
| Nbr_postdoc_years = 1 | 23,632 | 0.265 | 0.441 | 0 | 1 |
| Nbr_postdoc_years = 2 | 23,632 | 0.113 | 0.317 | 0 | 1 |
| Nbr_postdoc_years = 3 | 23,632 | 0.0597 | 0.237 | 0 | 1 |
| Nbr_postdoc_years = 4 | 23,632 | 0.0429 | 0.203 | 0 | 1 |
| Nbr_postdoc_years = 5 | 23,632 | 0.0196 | 0.139 | 0 | 1 |
| Humanities/Arts | 23,632 | 0.0156 | 0.124 | 0 | 1 |
| Social Sciences | 23,632 | 0.109 | 0.311 | 0 | 1 |
| Natural Sciences/Math | 23,632 | 0.519 | 0.500 | 0 | 1 |
| Medicine | 23,632 | 0.180 | 0.384 | 0 | 1 |
| Engineering | 23,632 | 0.177 | 0.382 | 0 | 1 |
| Female | 23,632 | 0.197 | 0.398 | 0 | 1 |
| Age | 23,632 | 36.24 | 2.755 | 26 | 49 |
| German | 23,632 | 0.967 | 0.180 | 0 | 1 |
| Apprenticeship | 23,632 | 0.193 | 0.395 | 0 | 1 |
| Years worked before graduation | 23,632 | 3.846 | 2.178 | 0 | 21.88 |
| Years worked after graduation | 23,632 | 5.270 | 1.024 | 0.334 | 6 |
| Years worked same operation | 23,632 | 2.955 | 1.708 | 0.0356 | 17.13 |
| Last employment in non-university research institute | 23,632 | 0.289 | 0.453 | 0 | 1 |
|  |  |  |  |  |  |
