# Supplementary material for: Postdoctoral employment and future non-academic career prospects
Source: PLoS One. 2022 Dec 1;17(12):e0278091. doi: 10.1371/journal.pone.0278091 (PMC9714870; doi:10.1371/journal.pone.0278091)
Supplement: S3 Table — (DOCX) [file pone.0278091.s003.docx]

Table S 3 Subsample analysis for doctorate recipients working at university or non-university research institute

|  | (17) | (18) |
| --- | --- | --- |
|  | Log  daily wage  (imputed) | Log  daily wage  (imputed) |
|  |  |  |
|  | Last employment at university | Last employment at non-university research institute |
|  | OLS | OLS |
| VARIABLES | Full Sample | Full Sample |
| Ref. Nbr_postdoc_years = 0 | - | - |
| Nbr_postdoc_years = 1 | -0.0586*** | -0.0256 |
|  | (0.0095) | (0.0162) |
| Nbr_postdoc_years = 2 | -0.1533*** | -0.0522*** |
|  | (0.0140) | (0.0184) |
| Nbr_postdoc_years = 3 | -0.1700*** | -0.0750*** |
|  | (0.0163) | (0.0200) |
| Nbr_postdoc_years = 4 | -0.2000*** | -0.0541*** |
|  | (0.0174) | (0.0209) |
| Nbr_postdoc_years = 5 | -0.2249*** | -0.0940*** |
|  | (0.0256) | (0.0271) |
|  |  |  |
| Constant | 4.8722*** | 4.8431*** |
|  | (0.0892) | (0.1516) |
|  |  |  |
| Individual controls | YES | YES |
| Work experiences controls | YES | YES |
| Graduation year dummies | YES | YES |
| Degree-granting university dummies | YES | YES |
| Regional controls | YES | YES |
| Dummies occupational field | YES | YES |
|  |  |  |
| Observations | 23,943 | 9,453 |
| R-squared | 0.157 | 0.166 |
|  |  |  |

Robust standard errors in parentheses

*** p<0.01, ** p<0.05, * p<0.1
