# Supplementary material for: Postdoctoral employment and future non-academic career prospects
Source: PLoS One. 2022 Dec 1;17(12):e0278091. doi: 10.1371/journal.pone.0278091 (PMC9714870; doi:10.1371/journal.pone.0278091)
Supplement: S4 Table — (DOCX) [file pone.0278091.s004.docx]

Table S 4 Robustness analysis for additional push and pull factors for staying in academia after graduation

|  | (19) | (20) | (21) | (22) | (23) | (24) | (25) | (26) | (27) | (28) |
| --- | --- | --- | --- | --- | --- | --- | --- | --- | --- | --- |
|  | High Wage  (dummy) | Log  daily wage  (imputed) | Daily wage  (imputed) | Daily wage  (censored) | Log  daily wage  (censored) | High Wage  (dummy) | Log daily wage  (imputed) | Daily wage  (imputed) | Daily wage  (censored) | Log  daily wage  (censored) |
|  |  |  |  |  |  |  |  |  |  |  |
|  | Logit | OLS | OLS | Cnreg | Cnreg | Logit | OLS | OLS | Cnreg | Cnreg |
| VARIABLES | Matched Sample | Matched Sample | Matched Sample | Matched Sample | Matched Sample | Full Sample | Full Sample | Full Sample | Full Sample | Full Sample |
| Ref. Nbr_postdoc_years = 0 | - | - | - | - | - | - | - | - | - | - |
| Nbr_postdoc_years = 1 | -0.3715*** | -0.0677*** | -21.5956*** | -8.5333*** | -0.0738*** | -0.3801*** | -0.0631*** | -18.6252*** | -9.1930*** | -0.0787*** |
|  | (0.0449) | (0.0117) | (3.8916) | (1.1451) | (0.0110) | (0.0418) | (0.0109) | (3.5868) | (1.0646) | (0.0102) |
| Nbr_postdoc_years = 2 | -0.5570*** | -0.1148*** | -35.5978*** | -13.5724*** | -0.1118*** | -0.5165*** | -0.1049*** | -31.8405*** | -12.9200*** | -0.1047*** |
|  | (0.0618) | (0.0159) | (5.0581) | (1.5107) | (0.0143) | (0.0556) | (0.0142) | (4.4697) | (1.3554) | (0.0127) |
| Nbr_postdoc_years = 3 | -0.9044*** | -0.2014*** | -54.6371*** | -21.7113*** | -0.1847*** | -0.6192*** | -0.1212*** | -34.0565*** | -14.8658*** | -0.1220*** |
|  | (0.0900) | (0.0238) | (7.5314) | (2.0744) | (0.0207) | (0.0627) | (0.0163) | (5.1089) | (1.4791) | (0.0143) |
| Nbr_postdoc_years = 4 | -0.8312*** | -0.1690*** | -44.2634*** | -21.6715*** | -0.1762*** | -0.7174*** | -0.1256*** | -33.3478*** | -16.7407*** | -0.1337*** |
|  | (0.0975) | (0.0250) | (7.8227) | (2.2839) | (0.0216) | (0.0692) | (0.0173) | (5.4518) | (1.5932) | (0.0149) |
| Nbr_postdoc_years = 5 | -1.2319*** | -0.2302*** | -61.6687*** | -26.5195*** | -0.2351*** | -0.9532*** | -0.1476*** | -35.0536*** | -19.6304*** | -0.1674*** |
|  | (0.1547) | (0.0393) | (10.8528) | (3.4032) | (0.0347) | (0.0956) | (0.0241) | (7.7619) | (2.0981) | (0.0205) |
| Third party funding by prof (defl.) | 0.0001 | 0.0002 | 0.0776 | 0.0303*** | 0.0003*** | 0.0006 | 0.0001 | 0.0330 | 0.0286*** | 0.0002*** |
|  | (0.0006) | (0.0002) | (0.0558) | (0.0099) | (0.0001) | (0.0005) | (0.0001) | (0.0469) | (0.0082) | (0.0001) |
| Nbr. prof. degree granting university | 0.0011* | 0.0001 | 0.0487 | -0.0142 | -0.0002 | 0.0006 | -0.0000 | 0.0087 | -0.0132 | -0.0002 |
|  | (0.0007) | (0.0002) | (0.0554) | (0.0141) | (0.0001) | (0.0005) | (0.0001) | (0.0452) | (0.0116) | (0.0001) |
| Unemployment rate in university region | -0.0122 | -0.0023 | -0.0850 | -0.5343 | -0.0050 | -0.0092 | 0.0001 | 1.0058 | -0.4975 | -0.0045 |
|  | (0.0206) | (0.0053) | (1.7205) | (0.4598) | (0.0043) | (0.0172) | (0.0045) | (1.4261) | (0.3938) | (0.0038) |
|  |  |  |  |  |  |  |  |  |  |  |
| Constant | -0.9778 | 4.7989*** | 3.3781 | 163.0908*** | 5.1061*** | -1.1884* | 4.7467*** | 4.5260 | 163.8644*** | 5.1103*** |
|  | (0.7516) | (0.1750) | (56.2010) | (10.7259) | (0.1023) | (0.6288) | (0.1452) | (44.8562) | (8.9854) | (0.0851) |
|  |  |  |  |  |  |  |  |  |  |  |
| Individual controls | YES | YES | YES | YES | YES | YES | YES | YES | YES | YES |
| Work experiences controls | YES | YES | YES | YES | YES | YES | YES | YES | YES | YES |
| Graduation year dummies | YES | YES | YES | YES | YES | YES | YES | YES | YES | YES |
| Degree-granting university dummies | YES | YES | YES | YES | YES | YES | YES | YES | YES | YES |
| Regional controls | YES | YES | YES | YES | YES | YES | YES | YES | YES | YES |
| Dummies occupational field | YES | YES | YES | YES | YES | YES | YES | YES | YES | YES |
|  |  |  |  |  |  |  |  |  |  |  |
| Observations | 14,236 | 14,236 | 14,236 | 14,236 | 14,236 | 20,176 | 20,176 | 20,176 | 20,176 | 20,176 |
| (Pseudo) R-squared | 0.130 | 0.149 | 0.096 | 0.040 | 0.140 | 0.135 | 0.145 | 0.092 | 0.041 | 0.144 |
|  |  |  |  |  |  |  |  |  |  |  |

Robust standard errors in parentheses

*** p<0.01, ** p<0.05, * p<0.1
